# Effect of after action review on safety culture and second victim experience and its implementation in an Irish hospital: A mixed methods study protocol

**DOI:** 10.1371/journal.pone.0259887

**Published:** 2021-11-18

**Authors:** Siobhán E. McCarthy, Theresa Keane, Aisling Walsh, Lisa Mellon, David J. Williams, Loretta Jenkins, Catherine Hogan, Cornelia Stuart, Natasha Rafter

**Affiliations:** 1 Graduate School of Healthcare Management, RCSI University of Medicine and Health Sciences, Dublin, Ireland; 2 Division of Population Health Sciences, Department of Epidemiology, RCSI University of Medicine and Health Sciences, Dublin, Ireland; 3 Department of Psychology, Division of Population Health Sciences, RCSI University of Medicine and Health Sciences, Dublin, Ireland; 4 Department of Geriatric and Stroke Medicine, RCSI University of Medicine and Health Sciences, Dublin, Ireland; 5 National Quality Assurance and Verification Team, Health Service Executive, Dublin, Ireland; 6 Division of Population Health Sciences, RCSI University of Medicine and Health Sciences, Dublin, Ireland; Florida International University, UNITED STATES

## Abstract

**Background:**

After Action Review is a form of facilitated team learning and review of events. The methodology originated in the United States Army and forms part of the Incident Management Framework in the Irish Health Services. After Action Review has been hypothesized to improve safety culture and the effect of patient safety events on staff (second victim experience) in health care settings. Yet little direct evidence exists to support this and its implementation has not been studied.

**Aim:**

To investigate the effect of After Action Review on safety culture and second victim experience and to examine After Action Review implementation in a hospital setting.

**Methods:**

A mixed methods study will be conducted at an Irish hospital. To assess the effect on safety culture and second victim experience, hospital staff will complete surveys before and twelve months after the introduction of After Action Review to the hospital (Hospital Survey on Safety Culture 2.0 and Second Victim Experience and Support Tool). Approximately one in twelve staff will be trained as After Action Review Facilitators using a simulation based training programme. Six months after the After Action Review training, focus groups will be conducted with a stratified random sample of the trained facilitators. These will explore enablers and barriers to implementation using the Theoretical Domains Framework. At twelve months, information will be collected from the trained facilitators and the hospital to establish the quality and resource implications of implementing After Action Review.

**Discussion:**

The results of the study will directly inform local hospital decision-making and national and international approaches to incorporating After Action Review in hospitals and other healthcare settings.

## Introduction

In healthcare, failure to learn is evidenced by the continued high rates of adverse events [1, 2] that have significant effects on patients, families and healthcare staff [3]. In Ireland, two national patient chart reviews have found that approximately one in eight admissions are associated with an adverse event with minimal improvement between 2009 and 2015 [4, 5]. Patients and their families are the first affected but there is a second victim: the healthcare providers who can be traumatised by the events [3]. Up to half of healthcare professionals experience a second victim impact at least once in their lifetime including anger, guilt, flashbacks, difficulty concentrating and increased risk of making further errors [6].

How organisations deal with these events and support the patients and staff involved is important. Having a well-developed safety culture of shared values, attitudes and patterns of behaviour regarding safety [7] can reduce adverse events [8, 9] and staff burnout [10]. Second victim recovery is also supported by active incident learning [7] and debriefing processes [6]. For example, studies in hospitals in the United States (US) [11] and China [12] have demonstrated that a culture of non-punitive response to errors is significantly associated with reductions in second victim distress while the provision of organisational support mediates the relationship between the two variables.

Debriefing techniques have been used to support team-based reflection and enhance safety culture in hospital settings [13]. Currently, various forms of debriefing are being used to address the patient safety and team adaptation challenges arising from the Covid19 pandemic [14]. After Action Review (AAR) is a non-hierarchical facilitated structured discussion of an event that enables teams to come to a shared mental model about what happened, why it happened and to identify learning and improvements [15]. It is a specific form of debrief of a team involved in an event. The methodology originated in the 1970s from the US Army as a means of supporting collective learning [16]. It is now a fundamental part of army culture and is conducted either formally or informally at soldier, crew, squad, platoon and company levels of the military [16]. Since then AAR has been adapted by US fire fighters [17], public health emergency responders [18] and hospital settings [19]. AAR is differentiated from other forms of debriefing by its structured use of multiple specific questions [20]. Guidance for other debriefing methodologies are more generic e.g. gather, analyse, summarise [20].

The use of trained facilitators is key to successful AARs [15]. In studies of US fire-fighters [15, 17, 21, 22], AAR has been shown to reduce individuals’ experience of ambiguity in relation to the causes of events [22] and promote satisfaction with the process [15]. Effective facilitator skills (e.g. offers equal opportunity to speak) and good AAR attendee behaviours (e.g. compliance with the ground rule of respect) were positively related to team and organisational safety culture [15, 21]. The frequency that AARs were held and the meeting quality (e.g. satisfaction with the meeting outcomes) moderated these relationships [15, 17, 21, 22]. Poorly conducted AARs were linked to increased blame [15]. Conversely, well conducted AARs were linked to incident learning [15] which in healthcare environments is likely to have a positive impact on second victim recovery [6].

AAR is included in the World Health Organisation (WHO) International Health Regulations as a methodology for learning and identifying follow-up actions after a national level public health response [23]. Between 2016 and 2019, over 60 AARs were conducted in WHO regions, primarily to support learning from epidemics and pandemics [24]. Therefore AAR is an important and relevant global tool for learning from the current COVID-19 pandemic [23]. Importantly, AARs are also performed in hospital settings both after emergency response [18, 19, 25] and to debrief after adverse events [26]. In the context of incident management, AARs are intended to be beneficial for identifying learning and improvement actions to prevent similar incidents reoccurring in the future [20].

### Irish national incident management framework

In the Irish health services, AAR is one of a number of approaches to incident review endorsed for use by the Health Service Executive (HSE) 2018 and 2020 Incident Management Frameworks [27]. The four AAR questions in the Framework [27] are:

1. What did you expect to happen?
2. What actually happened?
3. Why was there a difference between what you expected and what actually happened?
4. What can be learned?

AARs in the HSE Incident Management Framework are expected to facilitate timely explanations for service users, and are thought to enhance safety culture and staff healing from the impact of errors [28]. AARs are intended to enable short-term responses to patients and families and immediate improvement actions [28]. For very serious incidents, known as category 1 incidents within the Framework, a more detailed review may be also required. In other categories of incidents, AAR can be used on its own as a full response. The HSE advise that where AAR is used as an incident review methodology, a short AAR Report is required and is stored on the National Incident Management System. This includes the incident report number, a brief description of the event, the key learning and the actions agreed (see the HSE AAR Report Template in the article online S1 File). The HSE advise that the reports are confidential, anonymised and do not include names. The HSE also advise that AAR participants are informed about the report in advance of attending the review meeting and that all participants should have the opportunity to sign-off the report prior to it being finalised. The HSE also recommend that AAR is used informally for learning from positive (e.g. successful running of virtual clinic) and everyday routine events (e.g. end-of-shift debriefing) and a report is not required for these. Frequent conduct of informal AARs helps to establish team-based reflection as a norm within organisations and informal AARs are believed to help foster staff preparedness for participating in formal AARs, when patient safety incidents do occur [28]. For patient safety incidents, the HSE recommends that the AAR process is explained to patients and families and any issues or questions they raise are brought to the AAR meeting. The outcome of the review process is expected to be fed back at a meeting with the patient/family and they should be provided with a copy of the final report [28]. For incidents that involve patient harm, the HSE advise that prior to informing patients and families about an AAR meeting, the required disclosure of patient harm should have taken place under the HSE Open Disclosure policy, which is a separate process to AAR [28].

### Simulation based after action review facilitator training programme

Since 2018, approximately 500 healthcare staff across the Irish Health Services have been trained as AAR Facilitators as part of the implementation of the HSE Incident Management Framework. This training uses a 1.5 day simulation based programme (Table 1) co-designed by the HSE and the Graduate School of Healthcare Management at the RCSI. The programme builds on a one-day simulation based approach taken to AAR training by University College London Hospitals [29]. The training is delivered to cohorts of 16 participants comprising clinical and non-clinical staff at the RCSI Simulation Centre. The morning of Day One of the training emphasises the role of the AAR Facilitator in defining the ground rules (e.g. respect, no hierarchy, confidentiality) with a review group and asking the HSE AAR structured set of questions. In the afternoon, each participant conducts a fifteen minute AAR with three actors (simulated healthcare professionals). Following this, each participant receives feedback from a trainer and their peers. In the six week interval period until Day Two of the training, participants are expected to raise organisational awareness of AAR and to practise facilitating AARs. They are advised to do their first AAR on a positive event to increase their confidence with the approach. On Day Two participants share their experience of raising organisational awareness and of conducting AAR on incidents and events with their peers. The trained facilitators take some time to consider the next steps for AAR implementation at their healthcare organisation. They are requested to consider potential unit and organisation level implementation strategies and to together engage in change strategies that may involve a combination of peer to peer, bottom-up and top-down approaches.

**Table 1:** HSE/RCSI simulation based after action review facilitator trsaining programme.

**Day One**:
Participants learn about the context of AAR in the HSE Incident Management Framework and in everyday routine work. Participants partake in: Experiential learning about facilitation skills Four simulated AAR scenarios based on real-life events (conduct one, observe three) Self and peer feedback and receive facilitator, actor and video feedback.
**Six Week Interval Period**:
Participants raise organisational awareness of AAR.
Participants practice facilitating AARs.
**Day Two**:
Participants attend a half day reflective practice session to share learning with their peers.
Participants receive a certificate of completion.

It is thought that AAR can make a significant contribution to improving patient safety in Ireland and other countries. However, despite the inclusion of this debrief methodology in national [27] and international guidelines [23], there is little direct evidence of its effect on safety culture and second victim experience in healthcare [30, 31]. Nor do we know the enablers and barriers to successful implementation in healthcare organisations or how it is being used post training in Ireland or its resource implications.

The Irish safety Culture and After Action Review Experience (iCAARE) study aims to:

1. Measure the effect on safety culture and second victim experience before and 12 months after the introduction of AAR practice into a hospital setting.
2. Train selected staff as AAR Facilitators using a Simulation Based AAR Training Programme.
3. Explore enablers and barriers to implementation of AAR in the hospital setting.
4. Assess how AAR was implemented and the costs of implementation in a hospital setting.

## Materials and methods

### Study design

**Fig 1:**
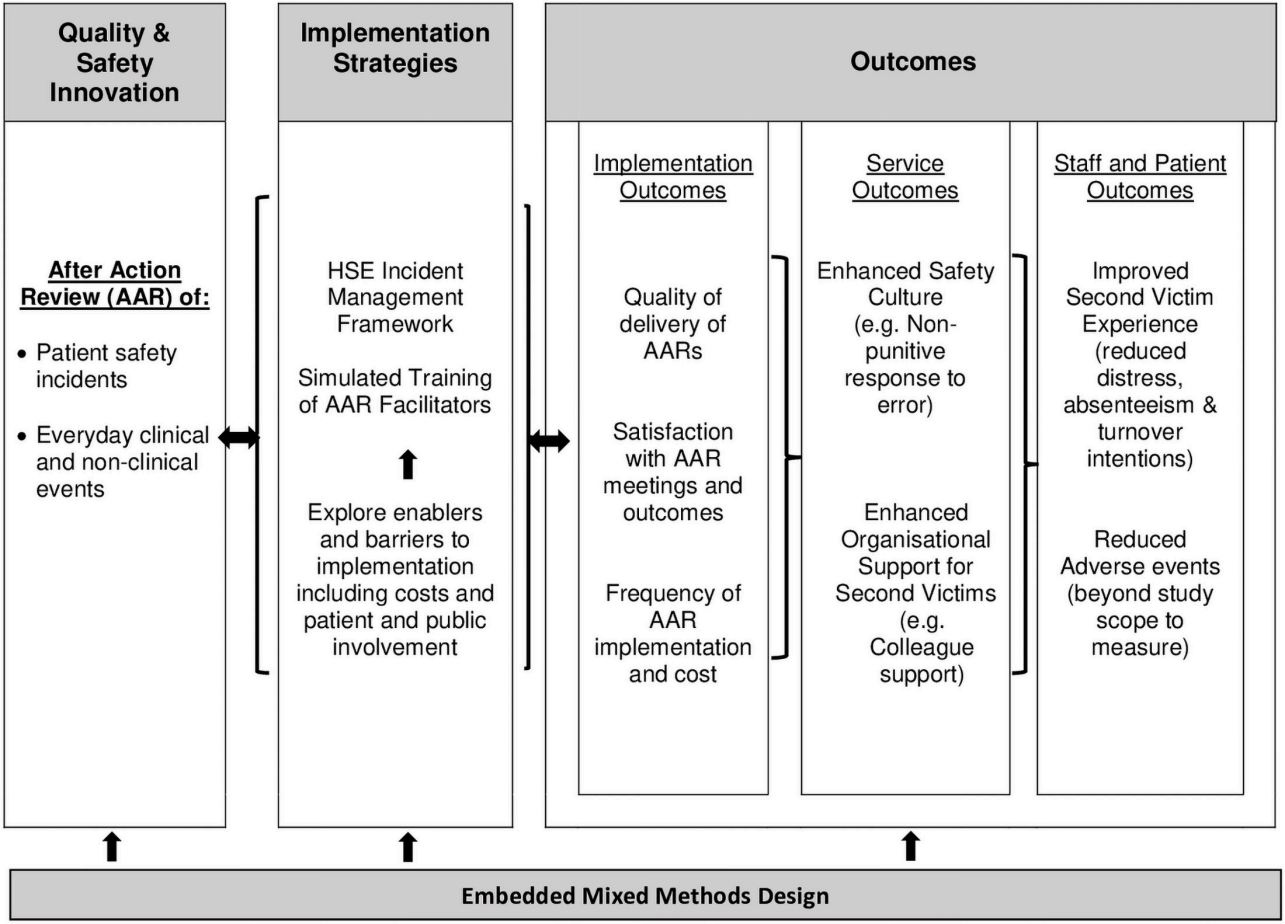
Study conceptual framework of effect of AAR on safety culture and second victim experience. Source: Conceptual Model adapted from Proctor et al. [33].

The iCAARE study will employ an embedded mixed methods design [32] to assess the effect and implementation of AAR in an Irish hospital. The dominant method used will be quantitative, supported by the collection of qualitative data. Quantitative methods will measure change in safety culture and second victim experience pre and post AAR implementation in the hospital. Staff views of the implementation will be examined using qualitative methods. The study conceptual framework (Fig 1) draws on a model of implementation advanced by Proctor and colleagues [33].

### Study site

The HSE and researchers will issue an expression of interest call to acute hospitals in the Irish Health Service. Criteria include that the hospital must be willing to (i) adopt the AAR approach, (ii) support staff in its use and (iii) have no staff already trained as AAR Facilitators. The Site Hospital Management will also conduct a readiness assessment for AAR with the HSE. This will support identifying the readiness of the hospital organisational culture to adopt AAR and to support staff to engage with it.

### Intervention

The study intervention is the introduction of a culture of AAR practice at the hospital site. The core implementation strategies designed to achieve this are the promotion and adoption of AAR as part of HSE National Incident Management Framework [27] and the training of hospital self-selected staff as AAR Facilitators. The training will be delivered by the Graduate School of Healthcare Management (authors TK and SMcC) at the RCSI Simulation Centre. It will be the standard HSE AAR training as described above.

### Participants

The study hospital site will self-select 1 in 12 staff to be trained based on standard guidance from the HSE (authors LJ and CH) on the candidate mix suitable for AAR Facilitator Training (e.g. clinical and non-clinical, spread across units/wards, multi-disciplinary). This sample size was selected as it was considered that at least 1 in 12 staff should be trained in order to generate a hospital wide culture of AAR. A site study gatekeeper, will provide those selected to be trained with an information leaflet about the study and they will be asked to complete a consent form to demonstrate awareness that the training is being provided as part of the iCAARE study. Following training, facilitators will be provided with a consent form to be contacted to participate in focus groups and an anonymous survey about AAR implementation.

### Data collection

#### Effectiveness of after action review on safety culture and second victim experience

*Instrument selection*. The study will use validated patient safety questionnaires: The Agency for Healthcare Research and Quality, Hospital Survey on Patient Safety Culture (AHRQ HSOPS v2.0) [34, 35] and Second Victim Experience and Support Tool (SVEST) [36–39].

The HSOPS 2.0 is a 32 item Likert type scale which measures 10 dimensions of safety culture in the work area, department, or clinical area of the hospital in which respondents spend most of their time. These dimensions include “teamwork”, “staffing and work pace”, “organisational learning”, “response to error”, “supervisor, manager or clinical leader support for patient safety”, “communication about error”, “communication openness”, “reporting patient safety events”, “hospital management support for patient safety” and “hand-offs and information exchange”. HSOPS 2.0 includes two outcome variables: “the number of events reported in the last twelve months” and “unit area patient safety rating”. Cronbach’s alphas for the 10 subscales of the HSOPS 2.0 ranged from 0.67 to 0.89 in 25 US Hospitals [40].

The SVEST comprises 29 items representing two outcome variables (absenteeism and turnover intentions) and seven dimensions. The seven dimensions measure two elements: second victim related distress (“physical distress”, “psychological distress” and “reduced professional self-efficacy”) and support following an incident (“colleague”, “supervisor”, “institutional” and “non-work-related” support). Most questions ask respondents to rate their agreement with statements on a 5-point Likert scale. Cronbach’s alphas spanned from 0.61 to 0.89 for the dimensions [36]. In addition, seven items which measure respondents preferred forms of support are included in the tool. Higher scores on both tools indicates a higher degree of perceived safety culture, of second victim distress and organisational support [11, 12].

*Sample*. At baseline, at least six weeks prior to AAR training, the HSOPS v2.0 and the SVEST will be administered together to all hospital staff. The expected response is a minimum of 30–50%.

*Survey administration*. The surveys will be administered online using SurveyMonkey. A study site gatekeeper will issue the link to surveys to staff emails accompanied with a study information leaflet. Participation is voluntary and the survey will be set up to be anonymous; the researchers will not know who has or has not responded. Follow-up reminders will be sent out and paper copies of surveys will also be made available to facilitate greater response. The invitation will be set up such that participants complete the HSOPS 2.0 first. This is designed to avoid priming participants to think about traumatic events from work (SVEST) which may then influence their perceptions of the hospital culture variables (HSOPS 2.0). The surveys have been effectively administered together in a similar fashion in prior studies [11, 12]. Twelve months after the AAR training, the questionnaires will be re-administered in the same way. The twelve-month safety culture survey will include additional questions about implementation of AAR in the hospital. These questions will be based on similar surveys of participants in AAR in other industries [21, 22] and will ask staff about their participation in AARs (formal and informal) over the previous 12 months—number and duration—and their satisfaction with the AAR process and outcomes.

#### After action review implementation

Implementation will be assessed using information from the focus groups and anonymous surveys of trained facilitators and additional items in the post safety culture follow-up survey of hospital staff. The resource implications of implementing AAR will also be collected from the hospital and HSE.

*Focus groups with the trained facilitators*. Focus groups will be conducted six months after facilitator training to explore enablers and barriers to implementation of AAR at the hospital. A study researcher will invite a stratified random sample (representative of different disciplines and genders) of trained AAR facilitators to participate. Each focus group will have six—eight number of people. Topic guides will be informed by the Theoretical Domains Framework [41] (a framework of influences on health professionals behaviour) to ensure applicability of findings to support implementation of AAR. The discussion will be audio recorded and transcribed.

*Survey of after action review facilitators*. An anonymous online survey using SurveyMonkey will be sent to trained facilitators twelve months after completion of training to establish how AAR is being implemented. The survey will be based on survey items used in a prior study of AAR Facilitators [21]. It will cover the number of formal and informal AARs each person has facilitated, their perceptions of participant behaviours and of their adherence to the process.

*Hospital incident reporting*. The number of incidents reported and the proportion with AARs at the hospital site over the study period will be obtained from the hospital’s Department of Quality and Safety.

*Resource implications*. Data will be collected on resource use in AAR implementation—e.g. staff education, facilitator training (time, materials) and AAR meetings. Total cost of implementing the AAR Training and the cost per AAR will be estimated in consultation with HSE and Hospital Site.

### Data analysis and mixed methods integration

#### Quantitative analysis

Analysis of HSOPS 2.0 and SVEST surveys will be performed using STATA version 16. The approach to survey data analysis will be adapted from a Chinese study examining the role of patient safety culture and organisational support in second victim distress [12]. For baseline data, the sum of the respondent positive response percentages (e.g. including only those with agree or strongly agree) from each item will be used to calculate the scales of HSOPS 2.0 and SVEST. The average positive response rate to safety culture dimensions in our HSOPS 2.0 will be compared with the most recent US AHRQ HSOPS database to benchmark the findings and identify significant differences. Baseline data will then be examined to establish if there is a link between patient safety culture, organisational support and second victim distress, including absenteeism and turnover intentions. At time two, the same analysis will be carried out as described above. Additionally, significant differences in major outcome variables (average positive response to safety culture dimensions, organisational support, average second victim distress and absenteeism and turnover intentions) between Time 1 and Time 2 will be compared using appropriate statistical techniques. The survey of AAR Facilitators will be analysed using descriptive statistics.

#### Qualitative analysis

Focus group transcripts will be entered into NVivo version 12. Framework analysis will be used to analyse data and to identify enablers and barriers to AAR [42]. It involves five iterative stages: familiarisation; identifying thematic framework; labelling; charting; mapping and interpretation [42]. One researcher will undertake the full five stages of analysis, with another researcher involved in double coding 30% of transcripts and applying the data to the Theoretical Domains Framework. The enablers and barriers identified will be mapped to the Theoretical Domains Framework [41] and to established behaviour change techniques to inform evidence based strategies for the implementation of AAR in Irish hospital settings (Table 2). A similar analytic approach was adopted in a qualitative study of barriers to nurses’ use of electronic medication management in two Australian hospitals [43].

**Table 2:** Approach to focus group analysis using the theoretical domains framework.

	Focus Group Analysis^a^
**Step One**	Identify enablers and barriers to participation in and practice of AAR
**Step Two**	Map individual enablers and barriers to the Theoretical Domains Framework
**Step Three**	Map behaviour change techniques to overcome key barriers and to optimise enablers for AAR participation and practice
**Step Four**	Propose interventions to operationalise relevant behaviour change techniques to address barriers and to optimize enablers to AAR

^**a**^ Analysis approach adapted from Debono et al. [43].

#### Data integration

Findings from each data collection process will be integrated to produce an overall study output. Integration will be carried out at the interpretation stage of the research [44]. A briefing sheet will be produced to synthesize the overall study findings. The study conceptual framework (Fig 1) will be used to develop themes and meta-themes. The findings about the implementation of AAR will therefore be used to help explain its effect on the study outcome variables. For example, findings on the frequency of AAR implementation and satisfaction will help explain the effect of AAR on safety culture and second victim experience. Conversely, findings from the surveys will be used to help explain enablers and barriers to implementation and implementation outcomes. For example, findings about the maturity of safety culture (e.g. response to error) may help explain the uptake and frequency of AAR.

### Data management

The use of a study gatekeeper means that no personal data about individuals will be processed by the Researchers without their consent. Questionnaires will be anonymous. Aggregated survey findings will be compared between Time 1 and Time 2, for overall responses. For the qualitative components of the study, codes (e.g. Participant 1) will be used to represent focus group participants. Findings will largely be presented as themes. No information that can identify individuals will be included in the study report or any publications.

All data will be stored on an encrypted folder on the RCSI Network. All the data sets will be stored securely for five years and then destroyed. Exceptions include audio recordings for focus groups which will be destroyed once data analysis has been completed.

### Ethical considerations

Few risks to the safety and well-being of participants is envisaged. Full study information leaflets will be provided to staff invited to participate in the research. Participation in all elements is voluntary. Those who do not wish to be contacted about the research, are assured at the outset that they can still undergo the training without any negative consequences. Written informed consent for all research elements (except anonymous questionnaires) will be obtained. Reflecting on adverse events however is a sensitive topic [45]. All study information leaflets emphasize that the contents of individual AARs are confidential to the review group and the researchers will not ask questions about individual events or reviews. To do this would impede staff comfort in participating in AARs. Rather study information leaflets assure that researcher questions about AAR will focus on general factors that enable or impede the AAR process at the hospital site (focus groups) and general uptake and satisfaction with the approach (surveys). At the same time, participation in surveys and focus groups about AAR, Safety Culture and Second Victim Experience may evoke memories of second victim trauma and of patient harm. The study information leaflets encourage staff to reach out to a trusted colleague for informal support and will provide the contact details of support agencies that provide services for staff struggling to cope with the impact of adverse events. The study received ethical approval from the RCSI University of Medicine and Health Sciences, Research Ethics Committee (REC202011024) on 19^th^ March 2021. Further ethical approval was received from the hospital site Research Ethics Committee on 21^st^ April 2021.

#### Patient and public involvement

As this study will provide benefits for both patient and staff groups, a patient and a staff advocate will be included in the study to inform the study implementation. Patients and families are not normally invited to participate in an AAR as this is primarily a mechanism for staff to de-brief in the aftermath of an incident [28]. The HSE’s Incident Management Framework [27] and accompanying guidance [28] however sets out the need to seek the involvement of service users and families in the incident management process. At a minimum this involves the need for the service to openly disclose incidents, advising the service user/family of the service’s plan for review, providing them with an opportunity to meet with the Review Team so that they can provide their perspective on the event and outline issues/questions that they would like addressed as part of the process and to be appraised on the outcome of the process. Any issues/questions raised by the service user/family may then be reflected in the Discussion at the AAR meeting. The key benefit for service users and families lies in the responsive nature of the approach thereby giving them information about the facts relating to the incident in a timely manner [28]. In the study setting, the hospital site will have full autonomy to decide how to involve patients and families in the AAR process. The study will collect data on how patients and families are being involved in the AAR process through the measurement and exploration of AAR implementation. The study findings will enable the hospital site to determine how well it is involving patients and families and develop strategies to enhance this.

### Study status

The iCAARE study incorporates researchers and knowledge users (persons in positions of authority to influence decisions on health policy and ensure findings of research are translated in their organisations). Both groups have been involved in co-developing the research questions and process and will work together to achieve the aims of the research. It is anticipated that the baseline surveys will commence in late spring 2021 and the AAR Training will be delivered in summer 2021.

## Discussion

### Contribution

The iCAARE study has the potential to make a real impact on establishing an evidence base for the effectiveness of AAR practice in healthcare environments both in Ireland and internationally. To our knowledge, this study is the first to examine the effect of AAR on safety culture and on second victim experience in a healthcare environment. Similarly, we are not aware of another longitudinal study that has, to date, utilised the HOPS 2.0 and SVEST together to examine impact of a patient safety intervention.

Fulfilment of the study aims will assist the HSE to decide how best to empower and engage staff to support patient safety. In healthcare settings, the study will contribute to addressing the conceptual hypothesis that AAR practice contributes to safety culture and staff well-being [27, 30]. Should AAR implementation enhance safety culture and reduce the impact of second victim experience, this may in turn reduce the likelihood of future errors to patients, and the risk of staff leaving clinical practice as a consequence of the effect of clinical incidents on them.

The examination of implementation, using the Theoretical Domains Framework will be beneficial to stakeholders. Theoretical approaches to identifying enablers and barriers to behaviour change and to designing targeted interventions have been shown to be more effective in changing behaviour than non-theory-driven approaches [46, 47]. The implementation findings will therefore support policy makers and healthcare providers to offer evidence based implementation strategies to enhance AAR usage. This will be particularly helpful for supporting team learning and improvement in the context of adverse events, routine work, and health system shocks, such as the Covid19 pandemic [48].

### Study limitations

It can be difficult to prove attribution in studies examining interventions to improve safety culture and second victim experience as multiple internal (e.g. other patient safety programmes) and external (e.g. national funding decisions and wholesale change to healthcare delivery during and after the COVID-19 pandemic) factors may also influence outcomes. Therefore the study will collect details on such influences and interpret findings accordingly. Another limitation may be a Hawthorne effect. Staff will be aware that their safety culture is being examined and could give more socially desirable survey responses [49]. However, the anonymity of survey participation may encourage comfort in responding honestly and offset socially desirable responses.

### Dissemination

The study results will be published in peer-reviewed journal articles and media releases. The media releases will target newspapers and staff magazines commonly read by patients, the public and healthcare staff. Dissemination will also take place in open access publications and at national and international conference presentations.

## Supporting information

S1 File HSE after action review report template. (DOC)
