# Supplementary material for: Effect of after action review on safety culture and second victim experience and its implementation in an Irish hospital: A mixed methods study protocol
Source: PLoS One. 2021 Nov 18;16(11):e0259887. doi: 10.1371/journal.pone.0259887 (PMC8601442; doi:10.1371/journal.pone.0259887)
Supplement: S1 File — (DOC) [file pone.0259887.s001.doc]

AAR Summary Report Template

The responses included in *italics* below are sample text only. Please replace with your own responses.

| **After Action Review Learning Report** |
| --- |
| NIMS Number: |
| Date of meeting: |

| Background to AAR |
| --- |
| *Provide a brief summary of the issue to which the AAR relates i.e. key chronological points here* |

| Key Learning Points Identified |
| --- |
| *Provide a brief summary of the learning points - these can be both items that worked well and those which could be improved. It is important to acknowledge both* |

| **Actions Agreed** |
| --- |
| *The actions agreed should be linked to the learning points identified above*  *1. Set out the actions agreed here*  *2. etc* |
